# The Plant-Associated Microbe Gene Ontology (PAMGO) Consortium: community development of new Gene Ontology terms describing biological processes involved in microbe-host interactions

**DOI:** 10.1186/1471-2180-9-S1-S1

**Published:** 2009-02-19

**Authors:** Trudy Torto-Alalibo, Candace W Collmer, Michelle Gwinn-Giglio

**Affiliations:** 1 Virginia Bioinformatics Institute, Virginia Polytechnic Institute and State University, Blacksburg, VA 24061, USA; 2 Department of Biological and Chemical Sciences, Wells College, Aurora, NY 13026, USA; 3 Department of Plant Pathology and Plant-Microbe Biology, Cornell University, Ithaca, NY 14853, USA; 4 Institute for Genome Sciences, University of Maryland School of Medicine, Baltimore, MD 21201, USA

## Abstract

All microbes that form beneficial, neutral, or pathogenic associations with hosts face similar challenges. They must physically adhere to and/or gain entry to host tissues; they must avoid, suppress, or tolerate host defenses; they must acquire nutrients from the host and successfully multiply. Microbes that associate with hosts come from many kingdoms of life and include bacteria, fungi, oomycetes, and nematodes. The increasing numbers of full genome sequences from these diverse microbes provide the opportunity to discover common mechanisms by which the microbes forge and maintain intimate associations with host organisms. However, cross-genome analyses have been hindered by lack of a universal vocabulary for describing biological processes involved in the interplay between microbes and their hosts. The Plant-Associated Microbe Gene Ontology (PAMGO) Consortium has been working for three years as an official interest group of the Gene Ontology (GO) Consortium to develop well-defined GO terms that describe many of the biological processes common to diverse plant- and animal-associated microbes. Creating these terms, over 700 at this time, has required a synthesis of diverse points of view from many research communities. The use of these terms in genome annotation will allow cross-genome searches for genes with common function (without demand for sequence similarity) and also improve the interpretation of data from high-throughput microarray and proteomic analyses. This article, and the more focused mini-reviews that make up this supplement to *BMC Microbiology*, describe the development and use of these terms.

## Introduction

Advances in sequencing technologies have accelerated the rate of whole-genome sequencing, resulting in the availability of full genome sequences for a diverse collection of microbes from many taxonomic groups. Among these are a large number of pathogens and other symbiotic organisms that live in close association with a host. The ability to query across these genomes offers the opportunity to uncover strategies shared by these organisms for overcoming the challenges faced in establishing and maintaining intimate associations with host organisms. However, effective use of these genome sequences to help understand host-pathogen interactions requires both structural and functional annotation, i.e. locating the genes as well as attaching meaningful information to them. In order for the functional annotation of individual genes to be maximally amenable to meaningful cross-genome searches, the vocabulary for describing the functions of gene products must be universally understandable across organisms. Traditional methods of attaching information to genes often fail to meet this requirement. For instance, gene names may be based on obscure mutant phenotypes rather than functionality of encoded gene products. In addition, genes that encode functionally equivalent proteins can have different names in different organisms. For example, XcpD, OutD, XpsD are various names for the outer membrane pore protein of the type II protein secretion pathway in different bacteria, and the type II secretion pathway itself is variously (and sometimes erroneously) known as "type II secretion", "the general secretion pathway", and "the main terminal branch" [1]. Another example is the "necrosis and ethylene-inducing protein", which was first reported from studies on *Fusarium oxysporium *and abbreviated as Nep1 [2]. Subsequently, homologs were identified in *Phytophthora *species and abbreviated as PsojNIP or NLP_Ps _in *P. sojae*, and NPP1 or NLP_Pp _in *P. parasitica *[3-5]. Finally, the same word sometimes means different things in different systems. An example is the term "sporulation," which can refer to both the reproductive sporulation process and the process that produces spores for survival during adverse environmental conditions, two very different biological processes.

A further problem with much existing genome annotation is that there is no way to tell which of many types of evidence has been used in assigning a particular annotation. For example, users of annotation data will find it valuable to know which annotations come from sequence-based approaches and which come from direct experimental confirmation using the annotated protein itself. Without such an evidence trail, it is impossible for users to evaluate the likely accuracy of the annotations they see in public resources.

The Gene Ontology Consortium (GOC) has addressed these limitations of traditional functional annotation. Representing an international collaboration, the GOC has developed, and continues to expand, a controlled vocabulary of terms arranged in three ontologies (molecular function, biological process, cellular component). These ontologies are currently being used to annotate gene products from a diverse set of species representing every kingdom of life [6]. In addition, the Gene Ontology (GO) effort has developed an extensive evidence tracking system which employs evidence codes to track the types of supportive information used for annotations [7].

Although quite comprehensive, the Gene Ontology as it existed in 2003 had limited terms for describing knowledge about biological processes involved in the interaction between microbes and their hosts. To meet this need, the Plant-Associated Microbe Gene Ontology (PAMGO) consortium [8] was formed in 2004 to develop GO terms that describe microbe-host interactions, in collaboration with the GOC. To create well-annotated reference genomes that provide high quality examples of the usage of the new terms, the consortium has been annotating the genomes of the bacteria *Pseudomonas syringae *pv *tomato *DC3000, *Dickeya dadantii *(*Erwinia chrysanthemii*) 3937, and *Agrobacteriun tumefaciens *C58; the fungus *Magnaporthe oryzae *(*M. grisea*); *and *the oomycete *P. sojae*.

## Scope of the PAMGO terms

**Figure 1:**
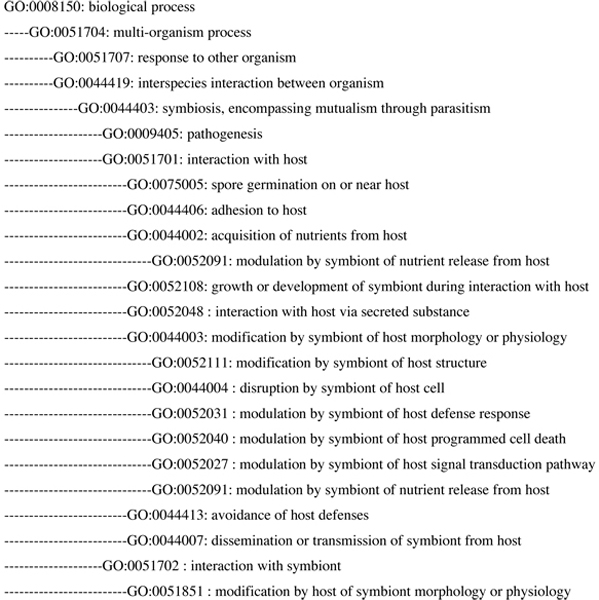
**Parent and child terms associated with " GO:0044403 symbiosis, encompassing mutualism through parasitism". ** "GO:0044403 symbiosis, encompassing mutualism through parasitism", was developed by the PAMGO consortium to emphasize the continuum of microbe-host relationships. "GO:0051701 interaction with host", a child term under GO:0044403, has several child terms that represent key processes in the interaction between diverse microbes and their host, irrespective of the symbiotic partner (mutualist or pathogen). To describe the fact that a particular symbiont-host association results in susceptibility, the term "GO:0009405 pathogenesis", a sibling of "GO:0051701 interaction with host", can be used.

The initial aim of the PAMGO consortium was to create terms associated with plant-pathogen interactions. However, it soon became apparent that creating more inclusive terms that were appropriate to both prokaryotic and eukaryotic microbes, to both plant and animal hosts, and for describing the whole range of intimate relationships between them (encompassing mutualism through parasitism), would better capture commonalities across diverse gene products involved in microbe-host interactions. After all, microbes of every domain face the same challenges in initiating an intimate association with a host. All must initially attach to the host and breach a barrier or enter through openings to gain access to a nutritional source; all must suppress, evade, or tolerate host defenses for successful invasion. In addition, it is known that microbes share strategies for invading a host, whether plant or animal. For example, bacterial pathogens of both plants and animals utilize the type III protein secretion machinery to inject effectors into host cells [9]. (Bacterial secretion systems, including the type III is reviewed in this supplement [10].) Some of those effectors target defensive signal transduction pathways common to both plant and animal hosts. Furthermore, pathogens as diverse as oomycetes (attacking plants) and protozoans (attacking animals) have been shown to share a common targeting domain in their secreted proteins that enter host cells [11,12]. Therefore we created an initial set of general terms to describe microbial activities common across the systems described above. Some of those general terms can be seen in Figure 1. In a different paper of this Gene Ontology-focused supplement, Lindeberg *et al*. [13] detail the GO annotation of type III effectors from both a plant pathogen, *Pseudomonas syringae *pv *tomato *DC3000 (PtoDC3000), and the animal pathogen *Escherichia coli*, emphasizing the similarities and differences in processes employed by these diverse pathogens in manipulating host defenses. A similar analysis reported in another paper in this series [14] extends the comparison to effectors of eukaryotic pathogens from diverse taxa, including oomycetes, fungi, and nematodes. The power of ontology-based annotation to capture common themes in such diverse pathogens is well illustrated in these two mini reviews.

## The continuum of symbiosis, encompassing pathogenesis through mutualism

Since the focus of PAMGO was initially on plant-pathogen interactions, one of the first challenges was to define the scope of a "pathogenic" interaction. Pathogenesis often includes the proliferation or reproduction of a microbe (e.g. bacterium, fungus, oomycete, nematode, protozoan) in a plant or animal host. The extent to which such proliferation and accompanying microbial processes are detrimental (and thus pathogenic) to the host depends on many factors present at the time, including the biotic or abiotic environment and the physiology of the host, especially the strength of the defense response. Also, the identical microbe or host process can be beneficial or detrimental depending on the context. For example, localized cell death associated with the plant defense response known as the hypersensitive response, which is effective against biotrophic and hemibiotrophic pathogens, can be considered beneficial to the host as a whole. The pathogen is curtailed at the point of infection and denied access to any living tissue at the necrotic front. On the other hand, for necrotrophs that live on exudates from dead tissues, the identical process of cell killing is beneficial to the pathogen. These examples illustrate the difficulties confronted by PAMGO and the GOC when considering whether newly developed GO terms that describe processes involved in pathogen-host interactions (e.g. "GO:0044406: adhesion to host") should be made "child" terms (i.e. sub-terms) of the existing GO term "GO:0009405: pathogenesis". Because such processes, even in the same microbe, might be part of initiating either a pathogenic or a more neutral interaction depending on the specific circumstances, we decided against such placement in the GO. Instead, we adopted "symbiosis" as a general term with its proper broad definition encompassing the whole spectrum of intimate relationships. The GO definition of this term notes "mutualism, parasitism, and commensalism are often not discrete categories of interactions and should rather be perceived as a continuum of interaction ranging from parasitism to mutualism." This definition also specifies that the word "host" refers to "the larger (macro) of the two members of a symbiosis," and that the word "symbiont" is used for "the smaller (micro) member." Accordingly, we adopted the word "symbiont" to designate the microbe in those GO terms that relate to microbe-host interactions. Once the broad definition of symbiosis had been accepted for use in the GO, the currently existing GO term "pathogenesis" became a child of "symbiosis," as did the general interaction terms such as "GO:0044406 adhesion to host" (Figure 1). Thus, if a specific microbial gene product is known to be involved in adhering to the host at the start of a pathogenic interaction, it can be annotated with the GO terms "GO:0009405 pathogenesis" and "GO:0044406: adhesion to host"; if it is instead a gene product involved in adhesion at the start of a mutualistic interaction, it can be annotated with GO terms for adhesion to host and mutualism.

## Addition of dual taxon capability to the Gene Ontology

The standard Gene Ontology annotation file has 15 fields to capture multiple types of information about the gene product being annotated [15,16]. Amongst these is one to capture the NCBI taxon id of the organism encoding the gene product. However, when annotating genes involved in interactions with other organisms, it is important to know not only the identity of the species from which the gene comes, but also the identity of the other organism that is involved in the interaction to which this gene product contributes. Capturing this information is especially important because the same microbial gene product can sometimes have one type of effect in one host species yet a different one in a different host (e.g. inducing vs. suppressing host programmed cell death (PCD)). Therefore, the specifications for the taxon field were modified to meet the microbe-host interaction community's need to capture the taxa of both organisms involved in a host-microbe interaction. Accordingly, the field now can accommodate two taxon ids, the first representing the organism encoding the gene product, and the second representing the organism with which the annotated organism is interacting. In cases where an effector protein secreted by a microbe triggers the hypersensitive response (HR) in a particular plant host, annotation of the microbial gene encoding the effector with GO term "GO:0034055 positive regulation by symbiont of host defense-related programmed cell death" would be accompanied by the taxon ids of both the microbe and the plant host. If the effector were shown to trigger the HR in two plant hosts, for example both *Arabidopsis *and soybean, there would be two separate annotations containing identical information except for the second taxon in the Dual Taxon field. Further discussion of PCD [17] and/or the dual taxon feature in GO [13,14] can be found in other articles in this supplement.

## Status of term development

There are currently over 700 GO terms that have resulted from the PAMGO effort. These include a set of very general terms describing the key processes involved in host-microbe interactions, including "adhesion to host", "acquisition of nutrients from host" (discussed in detail in this supplement by Chibucos and Tyler [18]) and "manipulation of host defenses". Also available are numerous child terms (i.e. sub-terms) that describe more specific processes. Most of the PAMGO-generated terms are found under the broad parent term "GO:0051704 multi-organism process" and its children terms "GO:0044419 interspecies interaction between organisms" and "GO:0051707 response to other organism" (Figure 1). A paper in this supplement [19] describes a recent development effort for GO terms, both general and specific, that describe processes involved in the interactions between eukaryotic pathogens and their hosts. In the GO, the more general terms usually describe processes that are shared across diverse organisms, while more specific terms are often created to describe organism-specific processes. For example one of the child terms of "GO:0044406 adhesion to host" is "GO:0052001 type IV pili-dependent localized adherence to host", a term relevant to bacterial symbionts. More recently added sibling terms to GO:0052001 include ones describing processes associated with adhesion of filamentous organisms to their host: "GO:0075001 adhesion of symbiont infection structure to host" and "GO:0075004 adhesion of symbiont spore to host" ([19] this supplement).

Since the focus of PAMGO was primarily on microbial pathogens, initial term sets were generated to annotate genes in the microbe that are involved in interactions with the host, e.g. "GO:0044405 recognition of host". However, it quickly became obvious that reciprocal terms that describe the interactions from the perspective of the host would also be required to meet all annotation needs (e.g. "GO:0051855 recognition of symbiont" Therefore, parallel sets of terms have been constructed to describe processes in the microbe as well as processes in the host that are involved in the interactions. In addition, terms were included to describe symbiotic relationships where neither organism could be clearly identified as "host" versus "symbiont." Thus, under the GO term "GO:0044419 interspecies interaction between organisms", there are child terms to accommodate symbiont genes that affect the host under "GO:0051701 interaction with host" and parallel terms appropriate for host genes under "GO:0051702 interaction with symbiont" (Figure 1). To learn more about these terms, including their definitions, synonyms, child terms, and genes annotated using them, see [20] and search using the term or a keyword within the term.

## Annotation of selected microbial genomes with new and existing GO terms

The members of the PAMGO consortium have been working on annotating the genomes of the bacteria *Pseudomonas syringae *pv *tomato *DC3000, *Dickeya dadantii (Erwinia chrysanthemii) *3937, and *Agrobacteriun tumefaciens*; the fungus *Magnaporthe oryzae (M. grisea)*; oomycete species. There are currently over 29,000 GO annotations as a result of the PAMGO project. The annotations can be viewed at [21]. As an example, Meng *et al*., [22] in this supplement report a comprehensive GO annotation of the rice pathogen *Magnaporthe oryzae*. In this paper, annotations were based on information from published literature as well as sequence-based analyses. Scientists studying these pathogens are encouraged to contribute annotations, and new terms where appropriate, to build on the work done so far by PAMGO consortium members. Please visit [23] for more information.

## Conclusion

A common set of terms to describe the activities of the gene products of pathogenic and beneficial microbes, as well as those of the organisms they affect, is a critical step toward understanding microbe-host-environment interactions. Use of a precise vocabulary for describing these genes in terms of their molecular functions, cellular locations, and biological processes, can facilitate discovery of underlying commonalities and differences involved in the interplay of diverse microbes with their hosts. In addition, these terms should be especially useful in the analysis of microarray and proteomics data produced in studies on host-microbe interactions. Ultimately, realization of the full power of GO depends on both the continuing development of new GO terms by the whole community to match the ever-increasing knowledge about host-microbe interactions, as well as increased usage of this resource by experimental scientists. While mastering any new language requires an initial investment, the potential for speaking directly, without translation, across all microbial genomes promises a commensurate payoff in future abilities to manipulate microbe-host interactions to our benefit.

## Competing interests

The authors declare that they have no competing interests.
